# N-ethyl-N-nitrosourea mutagenesis produced a small number of mice with altered plasma electrolyte levels

**DOI:** 10.1186/1423-0127-16-53

**Published:** 2009-06-08

**Authors:** Bernhard Aigner, Birgit Rathkolb, Martina Klempt, Sibylle Wagner, Dian Michel, Martin Hrabé de Angelis, Eckhard Wolf

**Affiliations:** 1 Chair for Molecular Animal Breeding and Biotechnology, Department of Veterinary Sciences, and Laboratory for Functional Genome Analysis (LAFUGA), Gene Center, LMU Munich, Munich, Germany; 2 Institute of Experimental Genetics, Helmholtz Zentrum München, Neuherberg, and Chair for Experimental Genetics, TU Munich, Freising-Weihenstephan, Germany

## Abstract

**Background:**

Clinical chemical blood analysis including plasma electrolytes is routinely carried out for the diagnosis of various organ diseases. Phenotype-driven N-ethyl-N-nitrosourea (ENU) mouse mutagenesis projects used plasma electrolytes as parameters for the generation of novel animal models for human diseases.

**Methods:**

Here, we retrospectively evaluated the use of the plasma electrolytes calcium, chloride, inorganic phosphorus, potassium and sodium in the Munich ENU mouse mutagenesis project where clinical chemical blood analysis was carried out on more than 20,000 G1 and G3 offspring of chemically mutagenized inbred C3H mice to detect dominant and recessive mutations leading to deviations in various plasma parameter levels.

**Results:**

We identified a small number of animals consistently exhibiting altered plasma electrolyte values. Transmission of the phenotypic deviations to the subsequent generations led to the successful establishment of mutant lines for the parameters calcium and potassium. Published data from other phenotype-driven ENU projects also included only a small number of mutant lines which were generated according to altered plasma electrolyte levels.

**Conclusion:**

Thus, use of plasma electrolytes detected few mouse mutants in ENU projects compared to other clinical chemical blood parameters.

## Background

Clinical chemical plasma analyses are often used in the medical examination of patients for the diagnosis of the involvement of various organs as well as for the evaluation of therapeutic strategies in multifactorial and polygenic human diseases. Electrolytes including calcium, chloride, inorganic phosphorus, potassium and sodium are routine parameters in these analyses. The diagnostic impact of plasma electrolyte values includes the general maintenance of osmotic pressure, water distribution and acid-base equilibrium (Na, Cl, K) as well as tissue-specific metabolism and organ function, especially of bone and kidney. Comparison of intracellular versus extracellular distribution of the electrolytes reveals that K is the chief intracellular cation, therefore, measured plasma K values are increased in the case of hemolysis or cellular stress like muscle trauma (Table 1). Together with the results of other diagnostic parameters, plasma electrolytes contribute to the identification of the impaired organ function(s) [1,2].

**Table 1:** Plasma electrolytes examined in the Munich ENU mouse mutagenesis project

Electrolyte	Intracellular vs. extracellular level	Diagnostic impact	Olympus kit/ISE	Range (mmol/l)^a^
Ca	Very low	Parathyroidea, bone, kidney	OSR6176	1–4

Cl	Low; major anion in plasma	Osmotic regulation, acid-base status, kidney	Ion-selective electrode	50–200

K	Major cation, low in plasma	K^+ ^transport system, acid-base status, kidney	Ion-selective electrode	1–10

Na	Low; major cation in plasma	Osmotic regulation, kidney	Ion-selective electrode	50–200

P, inorganic	Higher in cells	P uptake, bone, kidney	OSR6122	0.3–6.4

^a ^Linear measurement range (mmol/l) for the Olympus AU400 autoanalyzer (Olympus, Hamburg, Germany) and the reagents for human samples according to the manufacturer.

Biomedical research is done with mice as the animal models of choice and includes the search for alleles predisposing for or protecting against specific diseases. A strategy for the genome-wide generation and search of novel disease-related alleles consists of the random chemical mutagenesis of a large number of animals followed by systematic screening for clinically relevant disease phenotypes. The most widely used mutagen is N-ethyl-N-nitrosourea (ENU) which is mutagenic for premeiotic spermatogonial stem cells. This allows the production of a large number of randomly mutant offspring from treated males. ENU predominantly induces point mutations which results in allelic series for the functional analysis of genes [3]. During the last years, ENU mouse mutagenesis projects were established for the systematic, genome-wide, large-scale production and analysis of mouse mutants as model systems for inherited human diseases. They used appropriate routine procedures allowing the screening of large numbers of mice for a broad spectrum of parameters [4,5]. Mutant lines were established for various phenotypic parameters. ENU-induced mice with the causative mutation already identified are successfully used in different areas of biomedical research ([6,7] and refs. therein).

In the Munich ENU mouse mutagenesis project, a standardized screening profile of clinical chemical blood parameters was established for the analysis of offspring of mutagenized inbred C3H mice in order to detect phenotypic variants with defects of diverse organ systems and/or changes in metabolic pathways [8,9]. Here we retrospectively evaluated the generation of mutant lines exhibiting deviations from the physiological range of the plasma electrolyte values of Ca, Cl, K, Na and P.

## Methods

### Mutagenesis and breeding of mice

The experiments were carried out on the inbred C3HeB/FeJ (C3H) genetic background as described [4,10]. Ten-week-old male mice (= generation G0) were injected intraperitoneally with ENU (three doses of 90 mg/kg in weekly intervals).

The screen for dominant mutations was performed on G1 animals which were derived from the mating of the mutagenized G0 males to wild-type C3H females. Inheritance of the observed abnormal phenotype was tested on G2 mice which were derived from the mating of the affected G1 mouse exhibiting the altered phenotype and wild-type mice.

The screen for recessive mutations was carried out on G3 mice produced in a two-step breeding scheme from G1 mice. G1 males, which were excluded to exhibit dominant mutations by phenotypic analysis, were mated to wild-type females for the production of G2 animals. Subsequently, 6–8 G2 females were backcrossed to the G1 male to produce the G3 mice of the pedigree. The analysis of the inheritance of an observed abnormal phenotype in G3 mice was done on G5 mice. Therefore, the affected G3 mouse presumably harboring a homozygous recessive mutation was mated to a wild-type mouse for the production of the presumably heterozygous mutant G4 mice with an inconspicuous phenotype. Subsequently, the G5 mice derived from the intercross of G4 mice were tested for the abnormal phenotype. Alternatively, G5 mice derived from the backcross of a G4 mouse to the affected G3 animal were examined.

After the identification of the causative mutation, the provisional names of the established lines will be replaced according to the official nomenclature. Mouse husbandry was done under a continuously controlled specific pathogen-free (SPF) hygiene standard according to the FELASA recommendations [11]. All animal experiments were carried out under the approval of the responsible animal welfare authority (Regierung von Oberbayern).

### Clinical chemical analysis

Plasma from three-month-old G1 and G3 mice was analyzed for the electrolytes Ca, Cl, K, Na and P. In the cases where altered values below or above the physiologic range appeared, the mice were retested after three weeks. Blood samples were obtained by puncture of the retroorbital sinus under general short-term anesthesia. Plasma from Li-heparin treated blood was first analyzed for the electrolyte values using the Roche Hitachi 717 autoanalyzer (Roche, Mannheim, Germany) and the reagents for human samples (Roche), and in the second part of the project with the Olympus AU400 autoanalyzer (Olympus, Hamburg, Germany) and the reagents for human samples (Olympus) (Table 1). Calibration and quality control were performed according to the manufacturer's protocols.

In addition, the clinical chemical screen of plasma samples included substrates (cholesterol, creatinine, glucose, total protein, triglycerides, urea, uric acid) and enzyme activities (alanine aminotransferase, aspartate aminotransferase, alkaline phosphatase, α-amylase, creatine kinase) [12].

### Statistical analysis

The statistical analysis of the data was carried out using the software program Microsoft Excel 2000 (Microsoft, Redmond, WA). Values are presented as medians, and 95% and 90% ranges unless stated otherwise. Statistical significance (defined as p < 0.05) was evaluated using the χ^2^-test.

## Results and discussion

### Physiologic plasma electrolyte values in C3H mice

In the Munich ENU mouse mutagenesis project, the values for the plasma electrolytes Ca, Cl, K, Na and P were first determined in about 200 male and 200 female three-month-old wild-type C3H mice [13] and the 95% range of the values was defined to be physiologic [2]. According to the chosen physiologic range, abnormally low and high values were determined in the G1 and G3 offspring of ENU-mutagenized mice. The chosen physiologic range must allow to phenotypically detect animals with heritable defects leading to altered plasma parameter levels. On the other hand, it must exclude smaller variations from the mean level which are not suitable for breeding a mutant line over several generations according to the phenotypic variations. As the Munich ENU project was carried out in two different mouse facilities over a time period of many years, we re-evaluated the cut-off levels in retrospect for the present study. Plasma values of 15,700 to 16,600 G1 and G3 offspring of ENU-mutagenized mice were included for each electrolyte. Less than 1% of the G1 and G3 mice were expected to be primarily altered by ENU-induced mutations for a given blood parameter. Thus, the 95% range of the values determined in the large resource of animals should also comply with the successful detection and breeding of mutant animals with altered plasma electrolyte values. The analysis was done separately for the two time periods where different measurement equipments were used (Table 2). Calculation of the plasma electrolyte values for both mouse facilities separately and per year confirmed the results for both measurement equipments (data not shown). Comparison of the data of both equipments showed similar values for Ca, higher values for Cl, K and Na, and lower values for P with the Olympus equipment. Considerable sex-specific differences were seen for K, where males showed higher physiologic values (Table 2). Physiologic ranges of plasma electrolytes have been published from other projects [2]. Our data measured with the Olympus equipment are in accordance with published results which were determined with the same measurement equipment for three-month-old C3HeB/FeJ mice except of plasma Na where our data from the Roche equipment better fitted to the published results [14].

**Table 2:** Retrospective analysis of the physiologic range of plasma electrolytes (mmol/l) in three-month-old C3H mice of the Munich ENU project

		Roche measurement equipment^a^				Olympus measurement equipment^a^			
			
Electrolyte	Sex	n^b^	Median	95% Range^c^	90% Range	n^b^	Median	95% Range^c^	90% Range
Ca	m	6142	2.2	1.9–2.5	2.0–2.5	3945	2.2	1.8–2.4	2.0–2.4

Ca	f	4411	2.3	2.0–2.6	2.1–2.5	2152	2.2	2.0–2.5	2.0–2.4

Cl	m	6145	108	88–122	92–118	3345	115	104–126	106–124

Cl	f	4418	108	88–124	92–122	1836	115	102–125	106–123

K	m	6145	4.7	3.8–5.8	3.9–5.6	3345	5.0	4.0–6.0	4.2–5.8

K	f	4420	4.3	3.5–5.5	3.7–5.2	1836	4.6	3.8–5.8	4.0–5.6

Na	m	6146	152	138–164	140–162	3345	158	144–172	146–168

Na	f	4419	152	138–166	142–162	1836	156	144–172	146–168

P	m	6145	2.1	1.5–2.9	1.6–2.8	3923	1.8	1.4–2.6	1.4–2.4

P	f	4416	2.3	1.6–3.0	1.7–2.9	2151	2.0	1.4–2.6	1.4–2.6

m, male; f, female.

^a ^Plasma electrolyte levels were analyzed using the Roche Hitachi 717 autoanalyzer and the reagents for human samples, and subsequently the Olympus AU400 autoanalyzer and the reagents for human samples.

^b ^G1 and G3 offspring of ENU-treated mice for the dominant and recessive screen. About 60% of the analyzed mice were males.

^c ^Phenotypic variants were defined by two measurements of a three-week time period showing values below/above the lower/upper limit of the 95% range of the respective parameter.

Subsequently, deviations in plasma electrolyte values were defined in our analysis for mice showing values below or above the limits of the 95% range in two measurements of a three-week interval.

### ENU-induced phenotypic variants showing altered plasma electrolyte values

In the Munich ENU mouse mutagenesis project, more than 20,000 three-month-old G1 and G3 offspring of ENU-treated mice were screened for dominant and recessive mutations for various blood parameters. Plasma values of Ca, Cl, K, Na and P were detected in 15,700 to 16,600 G1 and G3 offspring. Table 2 shows the exact numbers of mice analyzed for each electrolyte. The examined offspring included 11,700 G1 mice and 6,300 G3 mice. For the five plasma electrolytes, 56 animals were identified as phenotypic variants showing decreased or increased values in two blood samples taken in a three-week interval (Ca low: 3 animals from the dominant screen/1 animal from the recessive screen, Ca high: 2/7, Cl low: 3/3, Cl high: 1/1, K low: -/-, K high: 7/12, Na low: 6/4, Na high: 2/1, P low: 3/6, P high: -/-). Six G1/G3 phenotypic variants showed deviations in two plasma electrolyte levels (three G1 and one G3 phenotypic variants for Cl and Na, two G3 phenotypic variants for Ca and Na). The 24 G1 phenotypic variants from the dominant screen of 11,700 G1 mice were offspring of 23 ENU-mutagenized G0 males. One G0 male produced two G1 phenotypic variants, one for Cl and Na, and the other for K. The 32 G3 phenotypic variants from the recessive screen of 6,300 G3 mice were offspring of 21 G1 males which were derived from 21 different ENU-mutagenized G0 males. Seven G1 males produced in total 18 G3 phenotypic variants (four G3 siblings with increased Ca, three G3 siblings with decreased P, three G3 siblings with altered Ca or P, three pedigrees each with two G3 siblings with increased K, and two G3 siblings with descreased Cl). Appearance of altered plasma electrolyte levels in several offspring of an ENU-mutagenized mouse may indicate a heritable mutation as cause for the affected phenotype allowing the establishment of a mutant line. In these cases, the phenotypic variants are presumed to harbor the identical causative mutation within the affected pedigree. However, only one of these affected pedigrees was analyzed for the transmission of the abnormal phenotype to the offspring resulting in mutant line KAL007 (see below).

For all five electrolytes, more than 80,000 measurements were taken. Having chosen the 95% range as physiologic data range, 100 animals of a wild-type population are calculated to show values below/above the cut-off level in two subsequent measurements by chance. This is twice the number of phenotypic variants identified with the plasma electrolytes as examination parameters in our ENU project. Plasma substrates and plasma enzyme activities as examination parameters detected higher numbers of phenotypic variants in the same resource of mice. 44 phenotypic variants were observed with increased plasma urea levels, more than 150 phenotypic variants showing hyper- or hypocholesterolemia, and more than 300 phenotypic variants were observed for the five plasma enzyme activities alanine aminotransferase, aspartate aminotransferase, alkaline phosphatase, α-amylase and creatine kinase ([15] and refs. therein).

### Establishment of mutant lines showing altered plasma electrolyte values

Phenotypic variants were tested for the inheritance of the deviations in plasma electrolyte values during the ENU project. This was analyzed on G2 offspring from the mating of G1 phenotypic variants to wild-type mice in the screen for dominant mutations, and on G4 × G3 backcross offspring after breeding G3 phenotypic variants to wild-type mice in the screen for recessive mutations. Sperm was cryo-preserved from male phenotypic variants which were not mated because of limitations of the breeding capacity wherever it was possible. A heritable altered plasma electrolyte level was diagnosed when offspring of the phenotypic variants showed values below/above the cut-off level in two measurements of a three-week interval.

During the ENU project, only few phenotypic variants with clearly altered plasma electrolyte levels were detected. Therefore, phenotypic analysis of the offspring was carried out only for six of the retrospectively identified G1/G3 phenotypic variants. One phenotypic G1 variant and one phenotypic G3 variant gave rise to no phenotypic mutant offspring. The other four phenotypic variants showed the inheritance of altered plasma electrolyte values to the offspring which included one phenotypic G1 variant showing decreased plasma Ca (line CA001) and three G3 animals showing increased K (lines KAL003, KAL004, KAL007). No sperm was cryo-preserved from line CA001. In addition, a mutant line showing increased K values (line KAL006) was produced by using a male G1 animal with the two measurements (5.8 and 5.7 mmol/l) below the cut-off level (Table 3). Establishment of further mutant lines with increased K values was not successful by breeding three additional G3 mice showing plasma K levels below the high cut-off level. In view of a putative renal cause for the increased plasma K values, the founder mice of all lines showed plasma urea levels within the normal range.

**Table 3:** Mutant lines showing deviations of plasma electrolyte values derived from the Munich ENU mouse mutagenesis project

				Altered plasma electrolyte values^b^: total (m/f)		
				
Phenotype	Mode of inheritance	Line	Offspring tested^a^: n (m/f)	n	% frequency	Mean (mmol/l)
Ca low	D	CA001	6 (2/4)	3 (2/1)	50 (100/25)	1.8/1.8

K high	D	KAL006	45 (26/19)	13 (10/3)	29 (38/16)	6.1/6.2

K high	R	KAL003	21 (14/7)	7 (5/2)	33 (36/29)	6.1/5.9

K high	R	KAL004	10 (5/5)	4 (2/2)	40 (40/40)	6.4/6.6

K high	R	KAL007	10 (5/5)	4 (3/1)	40 (60/20)	7.5/7.4

			10 (6/4)	6 (2/4)	60 (33/100)	7.6/7.3

m/f, males/females; D, dominant mutation; R, recessive mutation.

^a ^Offspring derived from mating heterozygous phenotypic mutants to wild-type mice (screen for dominant mutations) and after breeding homozygous mutants to heterozygous mutant mice (screen for recessive mutations). In addition, line KAL007 was successfully bred by mating homozygous mutants (second line).

^b ^The frequency of the altered phenotype in the offspring was expected to be 50% after mating heterozygous phenotypic mutants to wild-type mice (screen for dominant mutations) and after breeding homozygous mutants to heterozygous mutant mice (screen for recessive mutations). All offspring were expected to harbor the altered phenotype after breeding homozygous mutants of line KAL007 (second line). The absolute numbers of observed phenotypic mutants are given (n).

The frequency of the altered plasma electrolyte values in the five mutant lines was expected to be 50% after mating heterozygous mutants to wild-type animals in the lines with a dominant mutation and by breeding homozygous mutants to heterozygous mutant mice in the lines with a recessive mutation. The mutant phenotype was detected in more than half of the expected cases in all five lines. This indicated the monogenetic cause of the abnormal phenotype. In addition, this facilitates the effective subsequent phenotypic and molecular genetic analyses. In the lines with higher numbers of tested offspring, the numbers of animals exhibiting plasma K above the cut-off level were significantly (χ^2^-test, p < 0.001) higher than the expected 2.5% of the C3H mouse population when considering the 95% range as physiologic data range (Table 3).

Our previously published screen for altered plasma parameter levels resulted in the establishment of ENU-induced mutant lines showing changes of substrate (cholesterol, glucose, urea) and enzyme activity levels ([15] and refs. therein). In these ENU-induced mutant lines, there was a tendency of increased frequency of the altered phenotype with increasing age of the mice. Thus, the frequency of the mutant phenotypes may be underestimated in the present study as the mice were investigated at the age of three months. In addition, further in-depth analysis of our ENU-induced mutant lines often revealed higher frequencies of the altered phenotypes than observed in the long-term large-scale phenotyping project with the inherent impossibility of carrying out complete standardization [16]. The plasma electrolyte screen may be improved by analyzing the animals more frequently and/or at a higher age.

### Mutant lines showing plasma electrolyte deviations derived from other phenotype-driven ENU projects

Major centers performing phenotype-driven ENU mutagenesis-based projects used clinical chemical screens including plasma electrolytes as examination parameters for the generation of novel mutant mouse models [17]. Ca, Cl, K, Na and P were used in the CMHD ENU project , in the MRC ENU project [5,18] as well as in the RIKEN ENU project . The ENU projects used different genetic backgrounds: C57BL/6J × C3H , BALB/c × C3H [18], and C57BL/6J × DBA/2J or C57BL/6J × C3H .

Line 105-8-1 was described to show low plasma Ca values with low penetrance in a dominant mode of inheritance . Line EM:02158 (GENA383) showed high plasma Cl and Na values in a dominant mode of inheritance [18]. In addition, line EM:02163 (Candy5) was described to show hyperglycemia and high plasma P values , line MUTN/823.12.c (GENA/341) showed low plasma P and low plasma alkaline phosphatase activity, and line MUTN/941.6b showed low plasma high-density lipoprotein cholesterol and low plasma Na . Linkage analysis for the determination of the chromosomal site of the causative mutation was not yet described for any line. In the RIKEN ENU project, no mutant lines were published using Ca, Cl, K, Na and P as primary examination parameters in the clinical chemical analysis. However, the subsequent plasma electrolyte analysis in phenotypic mutants which have been recognized in other screens of the ENU project (e.g. using substrates and enzyme activities of the clinical chemical screen, blood pressure, behavior, dysmorphology) revealed deviations in the mutant mice .

Search for defined phenotypes in published chemically induced (ENU) mutants (as of 18.02.2009) of the "phenotypes and alleles" database in the Mouse Genome Informatics website  revealed 1947 alleles and 1606 genes/markers. Among them, 202 alleles and 172 genes/markers were described with phenotypes influencing "homeostasis/metabolism" and/or "renal/urinary" pathways, but no additional lines are described which have been revealed by the primary phenotype of plasma electrolyte deviations.

Plasma electrolytes are complementary parameters for the diagnosis of kidney and bone diseases. We recently described the generation of five ENU-induced mutant lines as novel kidney disease models according to their increased plasma urea levels [19]. The phenotypic mutant founders showed plasma electrolyte levels within the normal range. However, differences were observed between phenotypic mutants and wild-type littermate controls for defined plasma electrolytes in individual lines (unpublished results). Deviations of plasma Ca and P appeared in ENU-induced mutant lines which were established in dysmorphology screens for growth retardation and skeletal abnormalities (e.g. [20-22]). In addition, measurement of plasma copper levels as examination parameter was successfully carried out to generate novel ENU-induced mutant lines for the analysis of specific functional pathways ; .

In contrast to the low numbers of phenotypic variants by using plasma electrolytes, the clinical chemical screens carried out for the generation of novel mutant mouse models in ENU projects were successful with plasma substrates (e.g. cholesterol, glucose, urea) and plasma enzyme activities as examination parameters ([15] and refs. therein) [18]; .

## Conclusion

In this study, we retrospectively evaluated the use of the plasma electrolytes Ca, Cl, K, Na and P in the clinical chemical blood analysis of the Munich ENU mouse mutagenesis project to detect dominant and recessive mutations leading to deviations in plasma levels. Analysis of 11,700 G1 mice revealed 24 G1 phenotypic variants from 23 ENU-mutagenized G0 males, and analysis of 6,300 G3 mice revealed 32 G3 phenotypic variants from 21 G1 males which were derived from 21 different ENU-mutagenized G0 males. To date, five mutant lines showing altered plasma electrolyte levels were produced. The causes for the increased plasma K levels of the mutant lines established in our ENU project may be deviations in the K distribution/metabolism as well as factors leading to cellular stress e.g. of blood cells. In contrast to other plasma parameters used and despite a rather wide physiologic range of the values of at least some parameters e.g. Cl, the own results and published data from other phenotype-driven ENU projects revealed a small number of mice with deviations in plasma electrolyte levels. One reason may be a redundant regulation of plasma electrolyte levels in essential pathways like the maintenance of osmotic pressure and acid-base equilibrium leading to the masking of single mutations by compensatory effects of other genes. Alternatively, animals with major plasma electrolyte deviations may rarely survive until the examination time point used. Thus, plasma electrolytes as examination parameters in ENU projects have less impact for the establishment of novel mutant mouse lines for the functional kidney and/or bone analysis but are successfully used as secondary diagnostic parameters for other primary genetic defects.

## Competing interests

The authors declare that they have no competing interests.

## Authors' contributions

BA analyzed the data and wrote the manuscript. BR, MK, SW and DM carried out the clinical chemical blood analysis and the breeding of the mice. MHdA and EW designed the project. All authors read and approved the final manuscript.
